# Implementation of a Hybrid Cardiac Rehabilitation and Symptom Scoring System in Patients with Inappropriate or Postural Sinus Tachycardia Referred for Sinus Node Sparing Hybrid Ablation

**DOI:** 10.3390/jcm14165879

**Published:** 2025-08-20

**Authors:** Marta Kornaszewska, Aleksandra Wilczek-Banc, Anna Ratajska, Ewa Piotrowicz, Bartosz Szkaradek, Mariusz Kowalewski, Piotr Suwalski, Natalia Ogorzelec, Antoni Wileczek, Magdalena Zając, Michał Pastyrzak, Sebastian Stec

**Affiliations:** 1 Department of Cardiac Surgery and Transplantology, National Medical Institute of the Ministry of Interior and Administration, 02-507 Warsaw, Polandnatalia.ogorzelec@pimmswia.gov.pl (N.O.); smstec@wp.pl (S.S.); 2 Private Cardiology Practice, 33-394 Kleczany, Poland; 3 Cardiac Rehabilitation Hospital, 38-481 Rymanów Zdrój, Poland; olabanc@gmail.com; 4 Psychological Therapeutic and Research Center, Dr Jan Biziel University Hospital No. 2, 85-168 Bydgoszcz, Poland; aniarat@wp.pl; 5 Department of Humanization Medicine and Sexology, Collegium Medicum, University of Zielona Góra, 65-048 Zielona Góra, Poland; 6 Telecardiology Center National Institute of Cardiology, 04-628 Warsaw, Poland; epiotrowicz@ikard.pl; 7 Institute for Cardiovascular Science, CardioNeuroLab, CardioMedicum, 30-002 Cracow, Poland; fnszkaradekbartosz@gmail.com; 8 Cardio-Thoracic Surgery Department, Heart and Vascular Centre, Maastricht University Medical Centre, 6229 HX Maastricht, The Netherlands; 9 Department for the Treatment and Study of Cardiothoracic Diseases and Cardiothoracic Transplantation, IRCCS-ISMETT, 90127 Palermo, Italy; 10 Thoracic Research Center, Collegium Medicum, Nicolaus Copernicus University, Innovative Medical Forum, 85-067 Bydgoszcz, Poland; 11 National Medical Institute of the Ministry of Interior and Administration, Centre of Postgraduate Medical Education, 02-507 Warsaw, Poland; suwalski.piotr@gmail.com; 12 Panaceum-Med., Przemyska 24, 38-500 Sanok, Poland; 13 Department of Special Pedagogy and Speech Therapy, Faculty of Pedagogy, Kazimierz Wielki University, 85-064 Bydgoszcz, Poland; nzozermed@wp.pl; 14 Faculty of Physiotherapy, Jan Grodek State University in Sanok, 38-500 Sanok, Poland; michalpastyrzak@gmail.com; 15 Private Physiotherapy Michał Pastyrzak, 38-500 Sanok, Poland; 16 Elmedica EP-Network SKA, 26-110 Skarżysko-Kamienna, Poland

**Keywords:** inappropriate sinus tachycardia, postural orthostatic tachycardia syndrome, sinus node-sparing hybrid ablation, hybrid cardiac rehabilitation, implementation

## Abstract

**Background/Objectives**: Patients with inappropriate sinus tachycardia (IST) and postural orthostatic tachycardia syndrome (POTS) exhibit complex clinical profiles due to autonomic dysfunction. While sinus node sparing (SNS) hybrid ablation is emerging as a promising therapy, there are no established guidelines worldwide for post-procedure patient management and care is mainly based on telemonitoring. In contrast, our hybrid cardiac rehabilitation (HCR) program integrates inpatient care and home-based telerehabilitation. We aim to evaluate the implementation of the HCR program, patient acceptance and adherence, and the effectiveness of the Malmö POTS scoring system in monitoring disease progression and rehabilitation outcomes. **Methods**: Patients underwent a personalized HCR program after SNS. The program included early mobilization, psychological support, respiratory therapy, and structured exercise. Clinical outcomes were assessed using symptom burden (Malmö POTS score), ECG parameters, exercise duration, perceived exertion, and rehabilitation adherence. **Results:** All patients completed the inpatient phase, and 87% completed the home-based phase. In the early postoperative period, pericarditis, anemia, and benign rhythm disturbances were mild and self-limiting. The Malmö POTS score decreased from 65.3 to 25.7. Lower perceived exertion early in the program correlated with clinical improvement. At the 2-month follow-up, 81% of patients no longer met the clinical criteria for IST/POTS without the use of medications. The program was evaluated as safe, feasible, and well-tolerated, with high patient satisfaction. **Conclusions**: A well-organized hybrid cardiac rehabilitation program after SNS is feasible, safe, and well-tolerated in IST/POTS patients. The Malmö POTS score may support outcome monitoring. The integration of individualized training and telemedicine represents a promising development for patients post-SNS ablation. While this study demonstrates feasibility and potential benefits, further controlled studies are needed to evaluate its impact on long-term recovery and symptom control.

## 1. Introduction

Patients with inappropriate sinus tachycardia (IST) and postural orthostatic tachycardia syndrome (POTS) often present with complex clinical profiles [1,2], including multiple phenotypes and multi-organ abnormalities associated with cardiovascular autonomic dysfunction (CVAD). Therefore, their management should include comprehensive and long-term rehabilitation tailored to the patient’s capacity, particularly in individuals with orthostatic intolerance [3]. Pharmacological treatment is primarily based on ivabradine [4] and/or beta-blockers administered at optimized doses [2,5]. Additional recommendations include a high-salt diet (6–10 g/day) and increased fluid intake (2.5–3 L/day); using compression garments (covering large venous reservoirs in the lower limbs and the pelvic region); consuming small, low-carbohydrate meals; avoiding prolonged bed rest; performing counter-maneuvers to prevent syncope (e.g., leg crossing and muscle tensing); avoiding caffeine, alcohol, and excessive heat; and strengthening psychological and social support [6]. Patients with severe, pharmacologically resistant symptoms of IST/POTS require advanced diagnostic tools, such as standardized cardiovascular autonomic testing (CAT) and electrophysiological study (EPS), for accurate differential diagnosis and qualification for further treatment. Until recently, only patients with IST were referred for percutaneous catheter ablation (modification or complete ablation of the sinus node). According to the SUSRUTA registry, this procedure was associated with a 100% failure rate, a high risk of complications (e.g., phrenic nerve injury and superior vena cava stenosis), and the need for urgent or delayed pacemaker implantation in as many as 50% of patients in long-term follow-up [7]. Over the past decade, sinus node sparing (SNS) hybrid ablation has emerged as a promising alternative for IST, POTS, and overlapping IST/POTS syndromes [8]. Early outcomes are encouraging, demonstrating superior long-term efficacy and safety compared to conventional non-hybrid approaches. SNS is currently under global evaluation in the HEAL-IST trial and registry [9,10]. To date, no global guidelines, standardized rehabilitation programs, or routine care protocols have been established for this specific patient group. The approach to post-SNS in most countries is telemonitoring and telecare. In Belgium, for instance, patients undergo implantation of an Implantable Loop Recorder (ILR), followed by phone consultations and outpatient clinical check-ups [8,9]. Our approach went further by developing a hybrid cardiac rehabilitation (HCR) program that integrates both inpatient and home-based telerehabilitation. This study aims to evaluate the implementation, patient acceptance, and adherence to the HCR program and to assess the effectiveness of the Malmö POTS scoring system in monitoring disease progression and rehabilitation outcomes [11].

## 2. Materials and Methods

### 2.1. Patient Population

This study included consecutive patients who underwent SNS between September 2023 and December 2024 at the Department of Cardiac Surgery and Transplantology, National Medical Institute of the Ministry of the Interior and Administration in Warsaw. All participants had a confirmed diagnosis of IST or POTS. According to the 2015 ACC/AHA/HRS expert consensus statement [12] and the 2019 ESC guidelines [13], for IST, individuals presented with a resting sinus rhythm heart rate (HR) >100 bpm, a mean 24-h HR >90 bpm, and an excessive sinus rate response to minimal physical exertion; for POTS, subjects exhibited a sustained HR increase of ≥30 bpm (or ≥40 bpm in individuals under 19 years of age) within 10 min of standing, in the absence of orthostatic hypotension, and with chronic symptoms of orthostatic intolerance lasting ≥6 months. Patients were eligible if, in accordance with the definition, their symptoms persisted despite at least six months of optimized non-pharmacological treatment and if ineffectiveness or intolerance of at least two medications (e.g., beta-blockers, ivabradine, or their combination) was documented. In all cases, an electrophysiological study (EPS) was performed before SNS.

### 2.2. Study Protocol

**Figure 1:**
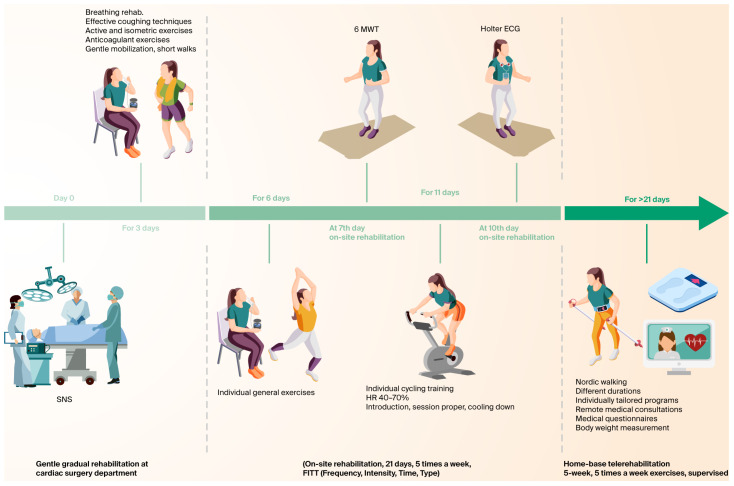
Timeline of the HCR protocol following SNS.

This study was approved by the University of Rzeszow’s Ethics Committee (decision number 5/4/2017-RARE-A-CAREgistry) and adhered to the Declaration of Helsinki. All participants gave their written informed consent. This study was designed as a prospective observational analysis in which patients underwent the SNS procedure. Immediately after the intervention, during hospitalization in the cardiac surgery department, early mobilization and clinical assessment were carried out. After discharge (typically on postoperative day 3), patients were referred to a specialized cardiac rehabilitation center for a 21-day inpatient rehabilitation program. At the beginning of this stage, a comprehensive clinical assessment was performed. Additional tests included complete blood count, C-reactive protein measurement, 24-h three-lead Holter ECG monitoring, transthoracic echocardiography, and ultrasound examination of the pleural cavities. Exercise training was conducted according to the FITT principle (Figure 1), determining the exercise dose in terms of frequency, intensity, time, and type. After 7 days, a 6-min walk test (6MWT) was performed, based on which patients were further qualified for training on a stationary cycle ergometer. The symptoms were assessed and monitored using the Malmö POTS score questionnaire system. In the next phase, patients participated in a 35-day home-based telerehabilitation based on Nordic walking, delivered via a hybrid smartphone platform. Before each session, patients measured their body weight, blood pressure, and oxygen saturation, performed a self-recorded ECG, and completed a symptom questionnaire. Different durations and tailored training were used. Sessions were ECG-monitored in real time to continuously assess heart rhythm and detect potential abnormalities that could occur after the procedure. After training, patients measured oxygen saturation and reported perceived exertion according to the modified Borg CR10 Category Ratio scale (0–10) (mBorg CR10). All data were automatically transmitted to the IT platform through a dedicated mobile application. Based on all collected measurements, together with the patient’s clinical condition and reported well-being, the supervising medical team individually adjusted the type and intensity of training. Sessions were delivered in either continuous or interval formats, depending on the patient’s exercise capacity and tolerance.

### 2.3. SNS Procedure 

SNS was performed in all patients using the single-stage hybrid approach in accordance with previous reports and the HEAL-IST protocol [9]. In short, a multidisciplinary team performed the ablation, with a cardiac surgeon accessing the heart via a minimally invasive right thoracoscopic intercostal approach and an electrophysiologist using a femoral or left jugular venous route. High-density 3D mapping of the right atrium and activation patterns during sinus rhythm (EnSite NavX/Precision™, Abbott St. Paul, MN, USA) was conducted before and after surgery. A bipolar bidirectional RF clamp (AtriCure EMR2) was used to isolate the superior and inferior vena cava at their junctions with the right atrium. Endocardial mapping and pacing maneuvers were performed to confirm conduction block, and additional endocardial ablation was applied at the operator’s discretion. Gentle, gradual mobilization as part of early recovery was initiated as early as the first postoperative day. After 3 days, participants were discharged with prophylactic ibuprofen, colchicine, and compressive stocks [14].

### 2.4. Program Protocol of Inpatient Rehabilitation

Inpatient rehabilitation based on the FITT principle was carried out under individual supervision. Exercise intensity was adjusted to the clinical condition and functional capacity of each patient [15,16]. The training process was divided into three stages: warm-up, main training, and cool-down [17]. The program began with gentle mobilization and breathing exercises, gradually progressing to general fitness exercises and interval endurance training on a stationary mini-cycle [16]. After seven days, the exercise capacity of stable patients was assessed using the 6MWT. In accordance with standard procedures, the test was performed between 10am and 12pm after the individuals had taken their regular medications. Markers were placed every 25 m, and patients performed a 6-min shuttle walk test. Subsequently, training sessions were conducted on a cycle ergometer, with individually adjusted workloads. The training heart rate was determined using the heart rate reserve method, which involves adding a specified percentage of the difference between maximal and resting heart rates to the resting heart rate, aiming to reach 40–70% of the heart rate reserve [16].

The program encompassed all aspects of cardiac rehabilitation care offered to patients, such as physical training, psychological support (including group and individual therapy), health education (lifestyle changes, nutrition, and physical activity), autogenic training, and individualized recommendations for physiotherapy. The primary goal was to optimize clinical status, improve physical fitness, prevent deconditioning, and restore functional independence.

### 2.5. Program Protocol of Home-Based Telerehabilitation

**Figure 2:**
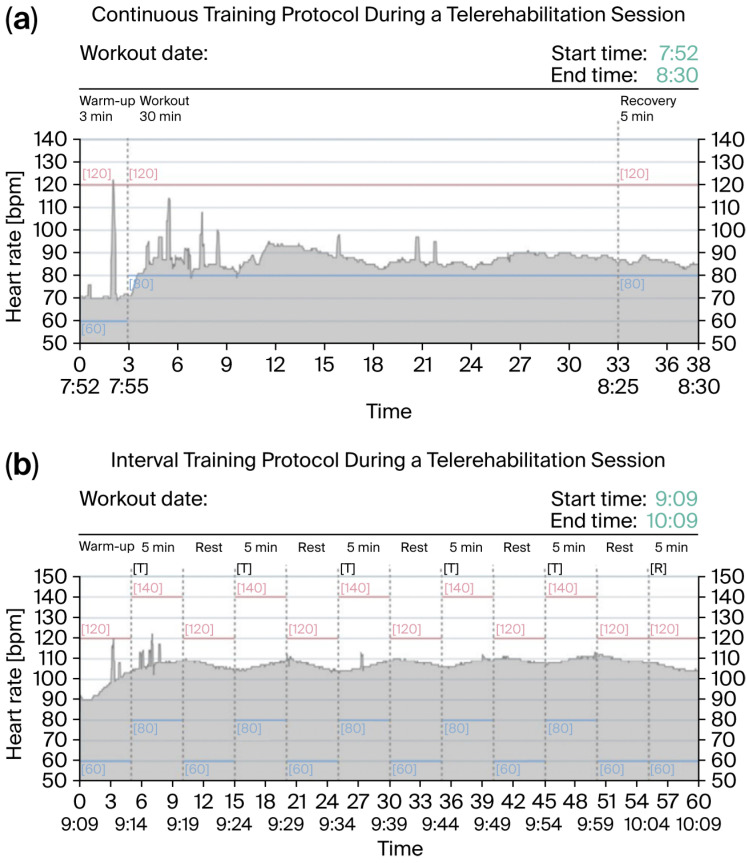
(**a**) Continuous training protocol of telerehabilitation session: commenced with a 3- to 5-min warm-up period, which was followed by 30 min of continuous exercise (target HR range 80–120 bpm), finishing with 5-min recovery period. Gray area—measured heart rate; colored horizontal lines—upper and lower HR limits (**b**) Interval training protocol during a telerehabilitation session: included alternating periods of 5-min exercises (target HR range: 80–140 bpm) and resting phases every other minute for the same duration. Session of 5 min exercise and 5 min rest phases, with the warm-up before, and cool-down following this interval session. Gray area—measured heart rate; colored horizontal lines—upper and lower HR margins.

**Figure 3:**
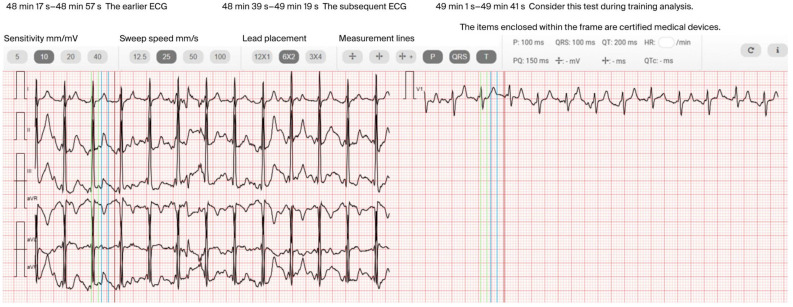
Continuous ECG monitoring performed with real-time data transmission to the online platform. Example of 49 min exercise recording showing continuous sinus rhythm at 136 bpm during training. The training data observed in real time is very high-quality and accurate, thus enabling precise patient monitoring and ensuring safety during training.

In the next stage, participants were referred to a telerehabilitation program based on a hybrid multi-recorder smartphone platform (DrEryk Kardio, Krakow, Poland). All sessions were monitored in real time by qualified medical personnel, enabling observation of the exercise course and immediate intervention in the event of symptoms. Telerehabilitation was implemented in the form of walking training via a smartphone platform, conducted as either continuous or interval walking (Nordic walking) [18,19]. Before each training session, the patient independently measured their blood pressure, body weight, and oxygen saturation; recorded an ECG using a wearable device; and completed a short pre-training questionnaire with the following questions: How are you feeling today? Have you taken all your medications? Do you have any symptoms of a cold or infection? All data were automatically transmitted to the telemedicine platform. The exclusion criteria for training were as follows: systolic blood pressure below 90 mmHg or above 160 mmHg, diastolic blood pressure below 50 mmHg or above 110 mmHg, an abnormal ECG recording (resting heart rate above 120 bpm, presence of conduction blocks, or arrhythmia), and feeling unwell or having symptoms of a cold. Continuous training sessions began with a 3- to 5-min warm-up and ended with a 5- to 10-min cool-down period (Figure 2a). In the interval training protocol, each session included alternating 5-min exercise and 5-min rest phases [17] (Figure 2b). All exercise phases were supervised by a programmed monitoring and control system capable of scheduling exercise intervals and transmitting ECG data [19] (Figure 3). Training was interrupted if HR fell outside the predefined range for each stage (80–120 and 80–140 bpm for continuous and interval training, respectively), or if the patient experienced malaise or alarming symptoms—in such cases, medical staff immediately contacted the subject. At the end of each session, the patient assessed the exertion performed using the mBorg CR10 and again measured oxygen saturation. These data were used to adjust the intensity of subsequent training sessions. The target exercise intensity was set at an mBorg CR10 value of 3, corresponding to moderate exertion. It is worth noting that, throughout the rehabilitation process, patients had the opportunity to participate in remote consultations with an electrophysiologist and a psychologist [20].

### 2.6. Clinical Assessment

On the second day of inpatient rehabilitation, echocardiographic examination and ultrasound of the pleural cavities were performed using a Philips Affinity 50G device. On the tenth day, a 24-h Holter ECG recording was obtained using a digital recorder (model DR 180+). To quantitatively assess symptom severity, the Malmö POTS scoring system questionnaire was used [10,11]. This questionnaire evaluates 12 of the most commonly reported symptoms, with a score from 0 (no symptom) to 10 (very severe symptom), yielding a total score range from 0 to 120 points. ECG monitoring and the active standing test were performed two months after completing the HCR program. The criteria for HCR effectiveness included both a reduction in the Malmö POTS score and the resolution of the clinical criteria for IST and POTS without pharmacotherapy. As the minimal clinically important change for the Malmö POTS scale has not yet been established, an improvement was defined as a score reduced below the validated diagnostic threshold of 42 points.

### 2.7. Psychological Support

Patients received comprehensive psychological care before the procedure, along with continuous access to remote consultations throughout the entire rehabilitation process [20].

### 2.8. Statistical Analysis

Statistical analysis of the collected data was limited to descriptive statistics, including frequency distributions (expressed as percentages), mean values, standard deviation, and confidence intervals. No formal hypothesis testing was conducted. All calculations were performed using Statistica, version 13.0 (TIBCO Software Inc., 2623 Camino Ramon Suite 200, San Ramon, CA, USA).

## 3. Results

During the EPS performed before SNS, slow–fast atrioventricular nodal reentrant tachycardia (AVNRT) ablation was carried out in seven cases (44%). Subsequently, SNS was successfully performed in all patients (100%) without significant early complications. Already on the first day of hospitalization, gentle and gradual mobilization was initiated, consisting of simple body weight exercises combined with stretching, breathing training, and strengthening exercises for small muscle groups. Within three days after the procedure, all patients were discharged from the hospital.

### 3.1. Implementation of Inpatient Rehabilitation and Early Clinical Events

**Figure 4:**
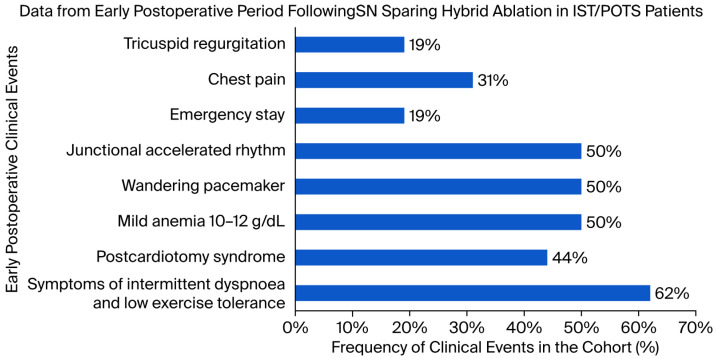
Frequency of early postoperative clinical events in a cohort of patients with IST/POTS undergoing sinus node sparing hybrid ablation. Data are presented as the percentage of patients in whom each of the described events was observed during the inpatient rehabilitation period. Reported events included tricuspid regurgitation, chest pain, unplanned emergency stay, junctional accelerated rhythm, wandering pacemaker, mild anemia (hemoglobin 10–12 g/dL), postcardiotomy syndrome, and intermittent dyspnea with low exercise tolerance. All conditions were self-limiting and did not result in serious complications.

All of the cohort (100%) finished inpatient rehabilitation that lasted 17.8 ± 4.2 days (mean ± STD). In the early postoperative period during inpatient rehabilitation, mild and transient symptoms of dyspnea and low exercise tolerance were observed in 10 out of 16 individuals (62%). In this group, unilateral and bilateral pleural effusions were present in five (50%) and three (30%) cases, respectively. Transient postcardiotomy syndrome was diagnosed in 9 out of 16 patients (44%) despite prophylactic colchicine administration in all cases (100%). The condition was self-limiting and did not lead to serious complications. Mild anemia (hemoglobin between 10 and 12 g/dL) was noted in eight cases (50%). Junctional accelerated rhythm and wandering atrial pacemakers were also observed in eight individuals (50%). Transient but echocardiographically significant tricuspid regurgitation (TR) was found in three patients (19%), including one case of severe TR (6%). Chest pain was reported by five cases (31%), with three individuals (19%) requiring additional consultations in the emergency department (emergency stay) due to the severity of symptoms; among them, two patients experienced panic attacks, and one reported chest pain caused by pleural effusion (Figure 4).

### 3.2. Efficacy of Inpatient Rehabilitation

In the second week of rehabilitation, all patients (100%) underwent 24-h ECG monitoring using the Holter method. In the analyzed group, the average HR was 77.0 ± 18.5 bpm (CI + 95% 67.22–86.91). The mean minimum HR was 58.2 ± 17.5 bpm (CI + 95% 48.88–67.49), while the mean maximum HR reached 106.6 ± 17.5 bpm (CI + 95% 96.36–114.76). Particular attention was given to reducing the average HR below 90 bpm, which was a key therapeutic goal. This target was achieved in 10 cases (62%). While not a primary outcome, achieving average HR control may indicate favorable physiological adaptation during rehabilitation.

### 3.3. Implementation and Acceptance of the Home-Based Telerehabilitation

Home-based telerehabilitation was implemented immediately after the inpatient phase in 14 individuals (87%). All participants accepted the standard guidelines for the home-based stage, which included Nordic walking training. The treatment and rehabilitation program was supervised by a highly qualified cardiologist. Throughout the study, patients also had the opportunity to consult their physician via phone or email at any time.

### 3.4. Adherence to Home-Based Telerehabilitation

The average number of planned training sessions was 20.14 ± 10.1 meetings (mean ± STD). Patients finished about an average of 14.8 ± 8.71 sessions, with dropout rates for personal issues and medical causes only amounting to 0.64 ± 1.08 and 0.28 ± 0.47 sessions, respectively. The number of sessions missed was an average of 5.28 ± 5.6.

During the first training session, participants with higher systolic blood pressure at baseline exercised for a shorter duration and achieved lower peak heart rates. Conversely, those with a higher resting heart rate tended to train longer, reach higher HR values during exercise, and achieve better post-exercise oxygen saturation. This may suggest that a higher baseline HR is associated with better early exercise tolerance, whereas elevated blood pressure may limit initial physical capacity. No relationship was observed between these baseline values and perceived exertion on the mBorg CR10 scores. Interestingly, already in the first week, those who ultimately achieved clinical improvement often rated their sessions as less demanding and reported lower mBorg CR10 scores in sessions 3 and 6. This may suggest that an early subjective perception of exertion could be, to some extent, related to later clinical outcomes. Overall, mBorg CR10 scores were similar regardless of the type of training.

### 3.5. Clinical Assessment

A noticeable improvement in reported symptoms was observed over the course of the study. The mean Malmö POTS score decreased from 65.3 before the SNS procedure to 25.7 after completing rehabilitation. At the two-month follow-up, normalized Malmö POTS scores and resolved clinical diagnostic criteria for IST and POTS were recorded in 13 individuals (81%) without pharmacotherapy (confirmed via ECG monitoring and the active standing test) [21,22].

### 3.6. Psychological Support

Individual psychological consultations were conducted before and/or after the SNS hybrid ablation procedure. During pre-surgery consultations, patients presented their previous history of symptom management and psychological adaptation to the disease dynamics and narrated their psychosocial functioning (partner and social relationships, work, and emotions). We focused on preparing the patients and their families for upcoming surgery. We mainly worked on anxiety, managing the emotions of patients and their closest ones, and communication issues within the family. The postoperative period is worth dividing into immediate (up to one month), short (up to six months), and longer (more than six months) periods. Immediately after the operation, patients experienced various emotions: joy that their surgery had been successful, anxiety about what would happen next, and fear of pain. The dynamics and intensity of emotions were high. In the short postoperative period, patients achieved initial emotional stabilization; cognitive–behavioral work was necessary for some of them. Usually, pain was managed as the patient had the necessary knowledge and skills. Sometimes, there was fear of symptoms or illness returning. Simultaneously, they felt the willingness and fear of returning to their daily routine, normal life, and work. Patients reported a sense of inner change and a need to redefine their personal identities. As such, the field of psychotherapeutic work emerged. In the distant postoperative period, the psychological adaptation of patients to the changing situation and maintaining a balance between challenges to their new abilities (including physical), anxiety, and family dynamics requires attention. These psychological experiences may have influenced how patients engaged in exercise and perceived their symptoms, which in turn could have contributed to the improvement observed in the Malmö POTS score.

## 4. Discussion

Our observational study is the first to show that the HCR model—combining inpatient rehabilitation with home-based telerehabilitation—is feasible, safe, and well-tolerated by patients with IST and POTS undergoing SNS. Upon completing the program, the vast majority of participants no longer met the diagnostic criteria for IST/POTS [23]. These findings are consistent with current perspectives presented by Taylor and Blakemore (2025) [24], who emphasize the role of home-based cardiac rehabilitation in improving accessibility and adherence. While these authors note that some meta-analyses indicate slightly greater effects with inpatient rehabilitation, they also point out that more recent data suggest no significant differences in treatment outcomes depending on the delivery model. Our results demonstrate that the hybrid model can be applied in this patient group; however, the absence of a control group limits the ability to draw definitive conclusions.

### 4.1. Clinical Implications of the Inpatient Rehabilitation Protocol

In our group, all patients successfully completed the inpatient rehabilitation program. In 14 individuals (87%), a composite endpoint of mild clinical events was recorded after SNS. Our observations highlight the need for close postoperative monitoring, which may yield better treatment outcomes. This partially aligns with the findings of de Asmundis et al. (2024), who demonstrated that prophylactic use of aspirin and colchicine significantly reduced the incidence of symptomatic pericarditis after SNS [14]. In their study, pericarditis occurred in 18.1% of patients receiving prophylaxis, compared with 52.8% in the group without such treatment. In our series, despite the widespread use of colchicine, postpericardiotomy syndrome occurred relatively frequently. This suggests that preventive strategies still require further refinement. In contrast to the meta-analysis by Aerts et al. (2024) [25], who evaluated the short-term outcomes of isolated and hybrid thoracoscopic ablation in patients with atrial fibrillation, no major cardiovascular events or deaths were observed in our population of individuals with IST/POTS, indicating a favorable safety profile of this procedure. Furthermore, early cardiac rehabilitation appeared to enhance safety with continuous monitoring of clinical symptoms in the postoperative period. Meanwhile, the combination of gradual mobilization with physiological parameter assessment was associated with improved physical performance. These findings highlight the need to educate cardiologists and rehabilitation specialists about the SNS procedure, potential postoperative complications, and appropriate responses in emergencies. Implementing remote consultations involving a multidisciplinary HEART-TEAM may further improve care coordination. However, further comparative studies are needed to more precisely define the risks and benefits of this approach in different patient groups, which would allow the development of individualized treatment strategies. It may also be worth considering dedicated training for cardiologists and rehabilitation teams on the specifics of post-SNS care.

### 4.2. Insights from the Home-Based Telerehabilitation Protocol

In this study group, implementing telerehabilitation proved to be safe and well-accepted. Most participants (87%) completed the program, with the few dropouts being mainly due to medical or personal reasons. Although no formal statistical analysis was performed, preliminary observations—particularly during the third and sixth sessions—showed that individuals who ultimately achieved clinical improvement often rated their sessions as less tiring. This may suggest that an early, subjective perception of lower exertion could be an indicator of later clinical improvement. The flexibility of the model, allowing for individual adjustments to training intensity and type, likely contributed to its positive evaluation by participants [26]. Based on our experience, the hybrid rehabilitation model appears feasible to implement and may serve as an effective form of support for patients after SNS for IST or POTS.

### 4.3. Interpretation of the Clinical Assessment

In our group, the Malmö POTS scoring system turned out to be a useful tool for quantitatively assessing symptom severity. The mean score decreased from 65.3 before rehabilitation to 25.7 after its completion. This result suggests that the scale may be valuable in monitoring treatment effectiveness. It is also worth noting that the intervention contributed to a reduced mean heart rate, and in most patients, the therapeutic target of <90 beats/min was achieved. In our study, the resolution of the diagnostic criteria for IST/POTS was considered an early indicator of clinical improvement, although other studies (e.g., HEAL-IST) have applied different definitions of remission or patient improvement. It should also be noted that although 62% of participants achieved the target mean heart rate, the wide range of obtained values indicates significant individual variability in response to therapy. This underlines the need to tailor the rehabilitation program to each patient’s profile and the importance of continuous heart rate monitoring with flexible adjustments to training intensity.

It should also be mentioned that not all patients may achieve complete remission without medication. As demonstrated in the HEAL-IST study, clinical improvement may occur later in the postprocedural period [9]. Symptom resolution can also be achieved by reintroducing pharmacological agents that were previously ineffective or poorly tolerated. Therefore, the absence of pharmacotherapy should not be the sole criterion for treatment success, and follow-up strategies should remain flexible, allowing for an individualized therapeutic approach.

### 4.4. Role of Psychological Support

A standardized psychological support program was introduced in cognitive behavioral methods with the assumption that in a post-surgical population, IST/POTS patients might show high levels of emotional reactivity and anxiety, particularly within early postoperative periods [27]. The organization of psychological care before and after the procedure most likely made it easier for patients to cooperate and be involved in rehabilitation. The findings emphasize the need for preoperative psychological support during recovery.

## 5. Limitations

Our study has several potential limitations. First, the sample size was relatively small and consisted of only women, which substantially limits both the statistical power and generalizability of the findings. However, it should be emphasized that the small number of participants reflects the early stage of SNS implementation in our region. For comparison, the only published experience from Central Europe to date includes only 20 patients (Stec et al., 2025) [28], which highlights the need for further regional research. We emphasize that this was a pilot study, initially designed to assess the feasibility, implementation, acceptance, and adherence of the HCR program. It is also worth noting that the sex distribution in our study reflects the epidemiological profile of IST/POTS, which predominantly affects younger women. Second, we acknowledge that the lack of a control group makes it difficult to precisely determine the extent to which the applied rehabilitation program contributed to the observed clinical improvement. Unfortunately, there is currently no standardized model of post-SNS care that could serve as a reference point. In most centers worldwide, post-procedure management solely relies on telemonitoring. In this context, our study offers a practical alternative care pathway and serves as a starting point for future multicenter studies with randomization or a control group. Third, in our group, there was only a single 6MWT, which does not allow objective evaluation of the physical performance in detection. However, it should be emphasized that the 6MWT was primarily used to determine the patient’s baseline functional capacity and to individualize the training program in our study. Finally, it should be noted that patient-reported outcomes are inherently subjective, which in itself constitutes a limitation. Nevertheless, the Malmö POTS scoring system used in this study was selected for its standardization and validation, as well as its ability to better capture the spectrum of symptoms typical of IST/POTS. These data were supplemented with objective measurements, including ECG monitoring, and the active standing test performed two months after completing the HCR program.

## 6. Conclusions

Our hybrid HCR program—combining inpatient rehabilitation with home-based telerehabilitation—proved to be feasible, safe, and fully acceptable in patients with IST and POTS after SNS and was characterized by a high level of adherence. In our group, short-term follow-up revealed markedly reduced symptom severity and improved physical performance, which was also reflected in decreased Malmö POTS scores and better heart rate control. Although these results may suggest the potential effectiveness of combining personalized, structured rehabilitation with telemedicine tools in this specific patient group, the absence of a control group ultimately prevents us from drawing definitive conclusions. Further prospective, multicenter, controlled studies are needed to confirm our observations and determine their long-term effects [29].

## Data Availability

Data are available from the corresponding author upon reasonable request. Due to privacy restrictions and the sensitive nature of patient data, the datasets generated and/or analyzed during the current study are not publicly available.

## References

[1] Mayuga K.A., Fedorowski A., Ricci F., Gopinathannair R., Dukes J.W., Gibbons C., Hanna P., Sorajja D., Chung M., Benditt D. (2022). Sinus Tachycardia: A Multidisciplinary Expert Focused Review. Circ. Arrhythm. Electrophysiol..

[2] Fedorowski A., Fanciulli A., Raj S.R., Sheldon R., Shibao C.A., Sutton R. (2024). Cardiovascular autonomic dysfunction in post-COVID-19 syndrome: A major health-care burden. Nat. Rev. Cardiol..

[3] Fu Q., Levine B.D. (2018). Exercise and non-pharmacological treatment of POTS. Auton. Neurosci..

[4] Abed H.S., Fulcher J.R., Kilborn M.J., Keech A.C. (2016). Inappropriate sinus tachycardia: Focus on ivabradine. Intern. Med. J..

[5] Ahmed A., Pothineni N.V.K., Charate R., Garg J., Elbey M., de Asmundis C., LaMeir M., Romeya A., Shivamurthy P., Olshansky B. (2022). Inappropriate Sinus Tachycardia: Etiology, Pathophysiology, and Management: JACC Review Topic of the Week. J. Am. Coll. Cardiol..

[6] Kanjwal K., Karabin B., Sheikh M., Elmer L., Kanjwal Y., Saeed B., Grubb B.P. (2011). Pyridostigmine in the treatment of postural orthostatic tachycardia: A single-center experience. Pacing Clin. Electrophysiol..

[7] Lakkireddy D., Garg J., DeAsmundis C., LaMeier M., Romeya A., Vanmeetren J., Park P., Tummala R., Koerber S., Vasamreddy C. (2022). Sinus Node Sparing Hybrid Thoracoscopic Ablation Outcomes in Patients with Inappropriate Sinus Tachycardia (SUSRUTA-IST) Registry. Heart Rhythm.

[8] de Asmundis C., Chierchia G.B., Lakkireddy D., Romeya A., Okum E., Gandhi G., Sieira J., Vloka M., Jones S.D., Shah H. (2022). Sinus node sparing novel hybrid approach for treatment of inappropriate sinus tachycardia/postural sinus tachycardia: Multicenter experience. J. Interv. Card. Electrophysiol..

[9] de Asmundis C., Pannone L., Lakkireddy D., Beaver T.M., Brodt C.R., Lee R.J., Frazier K., Chierchia G.-B., La Meir M. (2023). Hybrid epicardial and endocardial sinus node-sparing ablation therapy for inappropriate sinus tachycardia: Rationale and design of the multicenter HEAL-IST IDE trial. Heart Rhythm O2.

[10] Spahic J.M., Hamrefors V., Johansson M., Ricci F., Melander O., Sutton R., Fedorowski A. (2023). Malmö POTS symptom score: Assessing symptom burden in postural orthostatic tachycardia syndrome. J. Intern. Med..

[11] Fedorowski A. (2019). Postural orthostatic tachycardia syndrome: Clinical presentation, aetiology and management. J. Intern. Med..

[12] Page R.L., Joglar J.A., Caldwell M.A., Calkins H., Conti J.B., Deal B.J., Mark Estes N.A., Field M.E., Goldberger Z.D., Hammill S.C. (2016). 2015 ACC/AHA/HRS Guideline for the Management of Adult Patients With Supraventricular Tachycardia: Executive Summary: A Report of the American College of Cardiology/American Heart Association Task Force on Clinical Practice Guidelines and the Heart Rhythm Society. Circulation.

[13] Brugada J., Katritsis D.G., Arbelo E., Arribas F., Bax J.J., Blomström-Lundqvist C., Calkins H., Corrado D., Deftereos S.G., Diller G.P. (2020). 2019 ESC Guidelines for the management of patients with supraventricular tachycardia The Task Force for the management of patients with supraventricular tachycardia of the European Society of Cardiology (ESC). Eur. Heart J..

[14] de Asmundis C., Marcon L., Pannone L., Della Rocca D.G., Lakkireddy D., Beaver T.M., Brodt C.R., Monaco C., Sorgente A., Audiat C. (2024). Pericarditis prophylactic therapy after sinus node-sparing hybrid ablation for inappropriate sinus tachycardia/postural orthostatic sinus tachycardia. Heart Rhythm O2.

[15] Pelliccia A., Sharma S., Gati S., Bäck M., Börjesson M., Caselli S., Collet J.P., Corrado D., Drezner J.A., Halle M. (2021). 2020 ESC Guidelines on sports cardiology and exercise in patients with cardiovascular disease. Eur. Heart J..

[16] Piepoli M.F., Conraads V., Corrà U., Dickstein K., Francis D.P., Jaarsma T., McMurray J., Pieske B., Piotrowicz E., Schmid J.-P. (2011). Exercise training in heart failure: From theory to practice. A consensus document of the Heart Failure Association and the European Association for Cardiovascular Prevention and Rehabilitation. Eur. J. Heart Fail..

[17] Taylor J.L., Myers J., Bonikowske A.R. (2023). Practical guidelines for exercise prescription in patients with chronic heart failure. Heart Fail. Rev..

[18] Piotrowicz E., Zieliński T., Bodalski R., Rywik T., Dobraszkiewicz-Wasilewska B., Sobieszczańska-Małek M., Stepnowska M., Przybylski A., Browarek A., Szumowski Ł. (2015). Home-based telemonitored Nordic walking training is well accepted, safe, effective and has high adherence among heart failure patients, including those with cardiovascular implantable electronic devices: A randomised controlled study. Eur. J. Prev. Cardiol..

[19] Antoniou V., Davos C.H., Kapreli E., Batalik L., Panagiotakos D.B., Pepera G. (2022). Effectiveness of Home-Based Cardiac Rehabilitation, Using Wearable Sensors, as a Multicomponent, Cutting-Edge Intervention: A Systematic Review and Meta-Analysis. J. Clin. Med..

[20] Piotrowicz E., Mierzyńska A., Jaworska I., Opolski G., Banach M., Zaręba W., Kowalik I., Pencina M., Orzechowski P., Szalewska D. (2022). Relationship between physical capacity and depression in heart failure patients undergoing hybrid comprehensive telerehabilitation vs. usual care: Subanalysis from the TELEREH-HF Randomized Clinical Trial. Eur. J. Cardiovasc. Nurs..

[21] Grubb B.P., Kanjwal Y., Kosinski D.J. (2006). The postural tachycardia syndrome: A concise guide to diagnosis and management. J. Cardiovasc. Electrophysiol..

[22] Hou C.R., Olshansky B., Cortez D., Duval S., Benditt D.G. (2022). Inappropriate sinus tachycardia: An examination of existing definitions. Europace.

[23] Stec S., Suwalski P., De Asmundis C., La Meir M., Reichert A., Wilczek-Banc A., Szkaradek B., Kowalewski M. (2025). EP-Heart Team approach with sinus node sparing ablation for complex inappropriate sinus tachycardia and postural orthostatic tachycardia syndrome: A first experience in Central Europe. Kardiol. Pol..

[24] Taylor R.S., Blakemore A. (2025). Exercise-based cardiac rehabilitation: The importance of home-based approaches. Eur. Heart J..

[25] Aerts L., Kawczynski M.J., Bidar E., Luermans J.G.L., Chaldoupi S.-M., La Meir M., Kowalewski M., Maessen J.G., Heuts S., Maesen B. (2024). Short- and long-term outcomes in isolated vs. hybrid thoracoscopic ablation in patients with atrial fibrillation: A systematic review and reconstructed individual patient data meta-analysis. Europace.

[26] Jafri S.H., Guglin M., Rao R., Ilonze O., Ballut K., Baloch Z.Q., Qintar M., Cohn J., Wilcox M., Freeman A.M. (2023). Intensive Cardiac Rehabilitation Outcomes in Patients with Heart Failure. J. Clin. Med..

[27] El-Rhermoul F.-Z., Fedorowski A., Eardley P., Taraborrelli P., Panagopoulos D., Sutton R., Lim P.B., Dani M. (2023). Autoimmunity in Long Covid and POTS. Oxf. Open Immunol..

[28] Stec S., Suwalski P., de Asmundis C., LaMeir M., Kwaśniak E., Ogorzelec N., Reichert A., Wilczek-Banc A., Szkaradek B., Kowalewski M. (2025). Hybrid sinus node sparing ablation for complex inappropriate sinus tachycardia and postural orthostatic tachycardia syndrome: Initial experience in Central Europe. Pol. Arch. Intern. Med..

[29] Heindl B., Ramirez L., Joseph L., Clarkson S., Thomas R., Bittner V. (2022). Hybrid cardiac rehabilitation—The state of the science and the way forward. Prog. Cardiovasc. Dis..

